# Supplementary material for: Vortex clustering, polarisation and circulation intermittency in classical and quantum turbulence
Source: Nat Commun. 2021 Dec 7;12:7090. doi: 10.1038/s41467-021-27382-6 (PMC8651722; doi:10.1038/s41467-021-27382-6)
Supplement: Supplementary file 1 — Supplementary information. [file 41467_2021_27382_MOESM1_ESM.pdf]

Supplementary Information for Vortex clustering, polarisation and circulation intermittency in quantum turbulence

Juan Ignacio Polanco,[1,2] Nicolás P. Müller,[1] and Giorgio Krstulovic[1]

[1]*Université Côte d'Azur, Observatoire de la Côte d'Azur, CNRS, Laboratoire Lagrange, Boulevard de l'Observatoire CS 34229 - F 06304 NICE Cedex 4, France*

[2]*Univ Lyon, CNRS, École Centrale de Lyon, INSA Lyon, Univ Claude Bernard Lyon 1, LMFA, UMR5509, 69130 Écully, France*

(Dated: November 2, 2021)

I. TOY MODEL FOR VORTEX TANGLE POLARISATION AND CIRCULATION

In the main text, we have introduced a simple spin-like toy model for vortex polarisation based on a biased random walk. This model leads to different scaling laws for the circulation moments, which include Kolmogorov's 1941 theory (K41). We provide here more details on the analytical calculations.

The circulation of a loop enclosing n vortices is by definition

$$\Gamma_n = \sum_{i=1}^{n} s_i, \tag{1}$$

where $s_i = \pm 1$ is the polarization of the i-th vortex. As we have seen in the main text, the area A of the loop and the number of vortices can be related on average as $\langle n \rangle = \mathrm{A}/\ell^2$, with ℓ the mean inter-vortex distance.

The model is defined as follows. The signs of the set of vortices $\{s_i\}_{i=1}^{n}$ are chosen inductively in a random manner:

- $n = 1$: the sign of the first vortex, s_1, is chosen randomly with equal probabilities;
- $n > 1$: after computing the circulation Γ_n at step n, the sign of vortex $n+1$ is set to $s_{n+1} = 1$ with probability $\frac{1}{2} + \frac{1}{2} f\left[\frac{\Gamma_n}{n}\right]$, and $s_{n+1} = -1$ with probability $\frac{1}{2} - \frac{1}{2} f\left[\frac{\Gamma_n}{n}\right]$.

In order to ensure that the probabilities of the vortices are well defined, the function f must satisfy $-1 \le f[z] \le 1$ for $|z| \le 1$. Furthermore, to favour polarisation, i.e. to make it more likely for vortex s_{n+1} to be positive (resp. negative) if $\Gamma_n > 0$ (resp. $\Gamma_n < 0$), an additional condition is $f[z] > 0$ for $z > 0$ and $f[z] < 0$ for $z < 0$. Finally, as negative and positive circulation states are equally possible, one should have that $f[-z] = -f[z]$, and thus it suffices for f to be an odd non-decreasing function of z. Besides these general constrains, f can be arbitrary.

Clearly, in this model, the signs of all vortices are mutually correlated, but the circulation as a function of n is a Markov processes as $\Gamma_{n+1} = \Gamma_n + s_{n+1}$.

We denote by $\mathcal{P}_n(\Gamma)$ the probability that $\Gamma_n = \Gamma$. The master equation for $\mathcal{P}_n(\Gamma)$ can be directly computed using conditional probabilities

$$\mathcal{P}_{n+1}(\Gamma) = \sum_{\sigma=-\infty}^{\sigma=\infty} \mathcal{P}_{n+1}(\Gamma|\Gamma_n = \sigma)\,\mathcal{P}_n(\sigma) \tag{2}$$

$$= \mathcal{P}_{n+1}(\Gamma|\Gamma_n = \Gamma-1)\,\mathcal{P}_n(\Gamma-1) + \mathcal{P}_{n+1}(\Gamma|\Gamma_n = \Gamma+1)\,\mathcal{P}_n(\Gamma+1). \tag{3}$$

The infinite sum is reduced because $\mathcal{P}_{n+1}(\Gamma|\Gamma_n = \sigma)$ is the transition probability to a state where $\Gamma_{n+1} = \Gamma$, knowing that at the step n the circulation was σ. As circulation can only increase or decrease by one as an extra vortex is added, only two terms are non-zero. By construction of the model, we have for instance that $\mathcal{P}_{n+1}(\Gamma|\Gamma_n = \Gamma-1) = \frac{1}{2} + \frac{1}{2} f\left[\frac{\Gamma-1}{n}\right]$ as one needs a positive vortex to increase the circulation from $\Gamma - 1$ to Γ. It follows that

$$\mathcal{P}_{n+1}(\Gamma) = \left(\frac{1}{2} + \frac{1}{2} f\left[\frac{\Gamma-1}{n}\right]\right)\mathcal{P}_n(\Gamma-1) + \left(\frac{1}{2} - \frac{1}{2} f\left[\frac{\Gamma+1}{n}\right]\right)\mathcal{P}_n(\Gamma+1). \tag{4}$$

Multiplying by Γ^2 and summing over Γ we directly obtain an equation for the variance $\langle \Gamma^2 \rangle_n$

$$\langle \Gamma^2 \rangle_{n+1} = \langle \Gamma^2 \rangle_n + 1 + 2\left\langle \Gamma f\left[\frac{\Gamma}{n}\right]\right\rangle_n \tag{5}$$

In the simple case of a linear function $f[z]=\beta z$, one gets a closed recurrence equation that can be solved exactly. It is simpler to take the continuous limit in n, which leads directly to equation (3) of the main text. For a general function f, it is natural to assume that f can be developed in series as $f[z]=\sum_{i=0}^{\infty} f_{2i+1} z^{2i+1}$. It follows that

$$\frac{\mathrm{d}\langle\Gamma^2\rangle}{\mathrm{d}n} = 1 + 2f_1\frac{\langle\Gamma^2\rangle}{n} + 2f_3\frac{\langle\Gamma^4\rangle}{n^3} + \dots \tag{6}$$

We will show in the following that all the terms in the series but the one proportional to f_1 are subleading when n is large.

We express the circulation moments as

$$\langle\Gamma^p\rangle_n \sim a_p n^{\gamma_p}. \tag{7}$$

Inserting this Ansatz in Eq. (6) we obtain

$$a_2\gamma_2 = n^{1-\gamma_2} + 2f_1a_2 + \dots + 2f_{2p-1}a_{2p}n^{\gamma_{2p}-2p-\gamma_2+2} + \dots \tag{8}$$

Now, making use of Cauchy–Schwarz's inequality $\langle\Gamma^{2p}\rangle = \langle\Gamma^{2(p-1)}\Gamma^2\rangle \le \langle\Gamma^{2(p-1)}\rangle\langle\Gamma^2\rangle$, we have the general result for the scaling exponents $\gamma_{2p} \le \gamma_{2(p-1)} + \gamma_2 \le \gamma_{2(p-2)} + 2\gamma_2 \le \dots \le p\gamma_2$, for $p \ge 1$. We thus obtain

$$\gamma_{2p} - 2p - \gamma_2 + 2 \le p\gamma_2 - 2p - \gamma_2 + 2 = (\gamma_2 - 2)(p-1) \le 0, \tag{9}$$

since $\Gamma_n \le n$ and thus $\gamma_2 \le 2$. As a consequence, all the terms of the series but the first one are subleading. Now, if $\gamma_2 > 1$ then taking the limit of large n from Eq. (8) we have that $\gamma_2 = \min[2f_1, 2]$. On the other hand, if $\gamma_2 \le 1$ or equivalently $f_1 < 1/2$, all the terms depending on f can be neglected and we obtain $\langle\Gamma^2\rangle_n = n$, and thus $\gamma_2 = 1$.

For high-order moments, a similar analysis can be performed and yields at the leading order the equation

$$\frac{\mathrm{d}\langle\Gamma^p\rangle}{\mathrm{d}n} = 1 + pf_1\frac{\langle\Gamma^p\rangle}{n} + \dots. \tag{10}$$

In summary, for our model we obtain the self-similar exponents

$$\gamma_p = p\min\{\max[1/2, f'(0)], 1\}. \tag{11}$$

Note that performing the Laplace transform of (4), one obtains a linear partial differential equation that can be solved using the method of characteristics.

II. COARSE-GRAINED LOCAL ENERGY DISSIPATION AND ENSTROPHY FOR A DILUTED POINT-VORTEX GAS

Let us consider a two-dimensional system of n point vortices, each one carrying a positive or a negative circulation $\Gamma_i = \kappa s_i$, and located at the position $\mathbf{r}^i = (x^i, y^i)$. The corresponding vorticity field is given by

$$\omega(\mathbf{r}) = \kappa\sum_{i=1}^{n} s_i\delta_\xi(\mathbf{r}-\mathbf{r}^i), \tag{12}$$

where δ_ξ is regularisation of the two-dimensional Dirac δ-function at the scale ξ. Physically, ξ corresponds to the vortex core size where superfluid density vanishes or the Kolmogorov dissipative length scale in the case of classical turbulence. We assume that the system is diluted, meaning that the distance between vortices is much larger than their vortex core size $d^{ij} = |\mathbf{r}^i - \mathbf{r}^j| \gg \xi$.

To illustrate the spirit of the calculations, it is convenient to compute first the coarse-grained enstrophy at the scale r. By definition, this quantity is given by

$$\Omega_r = \frac{1}{\pi r^2}\int_{\mathrm{B}} \omega(\mathbf{r}')^2 \mathrm{d}^2\mathbf{r}' = \frac{\kappa^2}{\pi r^2}\sum_{i,j=1}^{n} s_is_j\int_{\mathrm{B}}\delta_\xi(\mathbf{r}'-\mathbf{r}^i)\delta_\xi(\mathbf{r}'-\mathbf{r}^j)\mathrm{d}^2\mathbf{r}', \tag{13}$$

where B is ball of radius r containing all the vortices. The integral is divergent when $\xi \to 0$ for $i=j$. Indeed, using for instance a Gaussian regularisation of the δ-function [1], we have $\int_{\mathrm{B}}\delta_\xi(\mathbf{r}'-\mathbf{r}^i)\delta_\xi(\mathbf{r}'-\mathbf{r}^j)\mathrm{d}^2\mathbf{r}' \approx \delta_{\sqrt{2}\xi}(\mathbf{r}^i-\mathbf{r}^j)$. As $d^{ij} \gg \xi$, the contribution of the integral vanishes for $i \ne j$, and therefore it behaves as $\sim \delta_{ij}/\xi^2$. Using this results, the coarse-grained enstrophy becomes

$$\Omega_r \approx \frac{\kappa^2}{\pi r^2}\sum_{i,j=1}^{n} s_is_j\frac{\delta_{ij}}{4\pi\xi^2} = \frac{\kappa^2 n}{4\pi^2\xi^2 r^2}. \tag{14}$$

Computing the coarse-grained energy dissipation field requires a bit more of work as one needs to use the gradient of the velocity field, but the same scaling with n and r will be obtained. The velocity field generated by the vortex i, evaluated at a distance $\Delta r_i = |\mathbf{r} - \mathbf{r}^i| \gg \xi$ is given by

$$\mathbf{v}^i(x, y) = \frac{\kappa s_i}{2\pi(\Delta r_i)^2}(y^i - y, x - x^i) \tag{15}$$

The total velocity field generated by n vortices is then $\mathbf{v}(x, y) = \sum_{i=1}^n \mathbf{v}^i(x, y)$.

The local dissipation of a viscous fluid is by definition

$$\varepsilon(\mathbf{x}) = \frac{\nu_{\text{eff}}}{2}\left(\frac{\partial v_i}{\partial x_j} + \frac{\partial v_j}{\partial x_i}\right)^2 = 2\nu_{\text{eff}}\left[(\partial_x v_x)^2 + (\partial_y v_y)^2 + \frac{1}{2}(\partial_x v_y + \partial_y v_x)^2\right], \tag{16}$$

with ν_{eff} an effective viscosity of the system that takes place at small scales. In the last equality we make explicit use that the system is two-dimensional. Using the velocity field given in Eq. (15), one obtains

$$\partial_x v_x^i = -\partial_y v_y^i = \frac{\kappa s_i n_x^i n_y^i}{\pi \Delta r_i^2} \tag{17}$$

$$\partial_x v_y^i + \partial_y v_x^i = \frac{\kappa s_i}{\pi \Delta r_i^2}\left[(n_y^i)^2 - (n_x^i)^2\right], \tag{18}$$

with $\mathbf{n}^i = (\mathbf{r} - \mathbf{r}^i)/\Delta r_i$. By replacing these expressions in Eq. (16), we obtain the local energy dissipation of a point-vortex system

$$\varepsilon(\mathbf{x}) = \nu_{\text{eff}}\left[4\left(\sum_i^n \frac{\kappa s_i n_x^i n_y^i}{\pi \Delta r_i^2}\right)^2 + \left(\sum_i^n \frac{\kappa s_i}{\pi \Delta r_i^2}\left[(n_y^i)^2 - (n_x^i)^2\right]\right)^2\right]. \tag{19}$$

The coarse-grained energy dissipation ε_r is defined by averaging $\varepsilon(\mathbf{x})$ on a disk B of radius r containing all the vortices

$$\varepsilon_r = \frac{1}{\pi r^2}\int_{\mathrm{B}} \varepsilon(\mathbf{r}')\mathrm{d}^2\mathbf{r}' = \frac{\nu_{\text{eff}}\kappa^2}{\pi^3 r^2}\sum_{i=1}^n\sum_{j=1}^n s_i s_j \int_{\mathrm{B}\setminus\cup_{k=1}^n \mathrm{B}(\mathbf{r}^k,\xi)} \frac{\left[(n_y^i)^2 - (n_x^i)^2\right]\left[(n_y^j)^2 - (n_x^j)^2\right] + 4 n_x^i n_y^i n_x^j n_y^j}{|\mathbf{r} - \mathbf{r}^i|^2 |\mathbf{r} - \mathbf{r}^j|^2}\mathrm{d}^2\mathbf{r}, \tag{20}$$

where $\mathrm{B}(\mathbf{r}^k, \xi)$ is a small ball of radius ξ around vortex $\mathbf{r}^k$ and we omitted primes on the integration variables to simplify notation. Those balls are excluded to avoid the divergences of point vortices and it is justified by the regularisation of the vorticity. As we will see, the integrals are dominated by such divergences.

For $i = j$, the integral is simpler and becomes

$$\int_{\mathrm{B}\setminus\cup_{k=1}^n \mathrm{B}(\mathbf{r}^k,\xi)} \frac{\left[(n_y^i)^2 - (n_x^i)^2\right]^2 + 4 n_x^i n_y^i n_x^i n_y^i}{|\mathbf{r} - \mathbf{r}^i|^4}\mathrm{d}^2\mathbf{r} \approx \int_{\mathrm{B}\setminus\mathrm{B}(\mathbf{r}^i,\xi)} \frac{\mathrm{d}^2\mathbf{r}}{|\mathbf{r} - \mathbf{r}^i|^4} \sim \frac{2\pi}{\xi^2}, \tag{21}$$

where we have used that $n_x^2 + n_y^2 = 1$, assumed that $r \gg \xi$ and kept only the dominant contribution. For $i \neq j$, the divergence is milder as in the denominator it intervenes the distance between two vortices d^{ij}, which is assumed to be much larger than ξ for a diluted system. Such terms contribute with a divergence of order $\log(\xi)/(d^{ij})^2$.

Finally, using Eq. (20) and keeping only dominant terms, we obtain at distances much larger than the inter-vortex distance

$$\varepsilon_r \sim \frac{\nu_{\text{eff}}\kappa^2}{\pi^3 r^2}\sum_{i=1}^n s_i^2 \frac{2\pi}{\xi^2} = \frac{2\nu_{\text{eff}}\kappa^2}{\pi^2\xi^2}\frac{n}{r^2} \sim \frac{\nu_{\text{eff}}\kappa^2 n}{\xi^2 \pi r^2}. \tag{22}$$

Note that in terms of scaling laws, we could have used Eq. (14) and $\varepsilon_r \sim \nu_{\text{eff}}\Omega_r$ to obtain the same result.

III. SUITABILITY OF OK62 THEORY FOR CIRCULATION SCALING EXPONENTS

The celebrated K41 theory for turbulence describes a self-similar behavior for the scaling exponents of the high-order moments of the velocity increments [2]. This theory can also be adapted to describe the scaling exponents of velocity circulation $\langle|\Gamma|^p\rangle \sim r^{\lambda_p}$, resulting in the scaling $\lambda_p^{\text{K41}} = 4p/3$. However, it was observed that the self-similar hypothesis breaks down

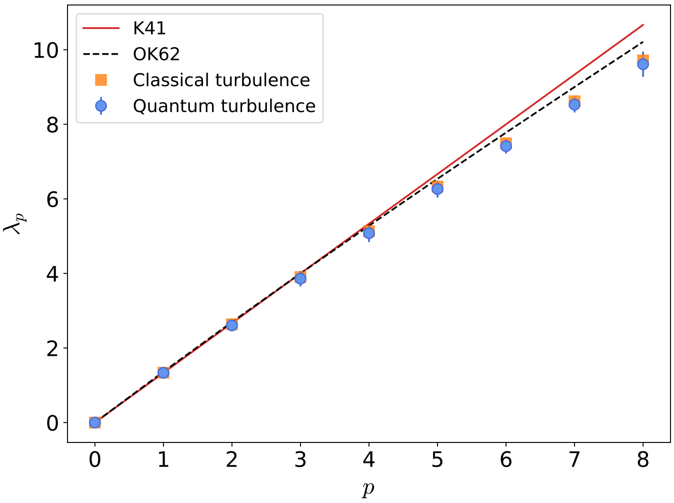

FIG. 1. Scaling exponents of the circulation moments defined as $\langle|\Gamma|^p\rangle \sim r^{\lambda_p}$ in numerical simulations of classical and quantum turbulence. As reference, the solid red line displays the Kolmogorov 1941 scaling $\lambda_p^{\mathrm{K41}} = 4p/3$ and the dashed black line shows the OK62 scaling defined in (23) using the She–Lévêque model for the scaling of dissipation (24). Error bars indicate 95% confidence intervals.

generating deviations in the scaling exponents, in particular for high-order moments. It was later proposed by Obukhov and Kolmogorov in 1962 a refined similarity hypothesis [3, 4], that can also be applied to the circulation exponents as

$$\lambda_p^{\mathrm{OK62}} = \frac{4p}{3} + \tau(p/3), \tag{23}$$

where $\tau(p)$ corresponds to the scaling exponents of the energy dissipation $\langle\epsilon^p\rangle_r \sim r^{\tau(p)}$. There are different models that describe the behavior of $\tau(p)$. In particular, in this work we use the She–Lévêque model [5]

$$\tau(p) = -\frac{2p}{3} + 2\left[1 - \left(\frac{2}{3}\right)^p\right], \tag{24}$$

that has no adjustable parameters.

Figure 1 shows the scaling exponents λ_p of the velocity circulation for moments up to order 8 obtained from numerical simulations of classical and quantum turbulence. Both numerical simulations were performed using 2048^3 collocation points, with a $Re_\lambda = 510$ in classical turbulence and a scale separation of $L/\xi = 1365$ in quantum turbulence. For high order moments, the scaling exponents deviate from both K41 and the refined OK62 models. These deviation were also observed in low-Reynolds numbers numerical simulations of the Navier–Stokes equation using the log-normal model for the scaling of dissipation [6].

---

[1] $\delta_\xi(\mathbf{r}) = \frac{1}{2\pi\xi^2} e^{-\frac{|\mathbf{r}|^2}{2\xi^2}}$.
[2] U. Frisch, *Turbulence: The Legacy of A.N. Kolmogorov*, 1st ed. (Cambridge University Press, 1995).
[3] A. M. Oboukhov, Some specific features of atmospheric tubulence, J. Fluid Mech. **13**, 77 (1962).
[4] A. N. Kolmogorov, A refinement of previous hypotheses concerning the local structure of turbulence in a viscous incompressible fluid at high Reynolds number, J. Fluid Mech. **13**, 82 (1962).
[5] Z.-S. She and E. Lévêque, Universal Scaling Laws in Fully Developed Turbulence, Phys. Rev. Lett. **72**, 336 (1994).
[6] N. Cao, S. Chen, and K. R. Sreenivasan, Properties of Velocity Circulation in Three-Dimensional Turbulence, Phys. Rev. Lett. **76**, 616 (1996).
